# Arginine Depletion in Human Cancers

**DOI:** 10.3390/cancers13246274

**Published:** 2021-12-14

**Authors:** Devi D. Nelakurti, Tiffany Rossetti, Aman Y. Husbands, Ruben C. Petreaca

**Affiliations:** 1 Biomedical Science Undergraduate Program, The Ohio State University Medical School, Columbus, OH 43210, USA; nelakurti.1@buckeyemail.osu.edu; 2 Biology Undergraduate Program, The Ohio State University, Marion, OH 43302, USA; rossetti.40@buckeyemail.osu.edu; 3 Department of Molecular Genetics, The Ohio State University, Columbus, OH 43215, USA; 4 Department of Molecular Genetics, The Ohio State University, Marion, OH 43302, USA; 5 Cancer Biology Program, The Ohio State University James Comprehensive Cancer Center, Columbus, OH 43210, USA

**Keywords:** mutation, arginine, purifying selection, cancer

## Abstract

**Simple Summary:**

Thousands of cancer genomes are now publicly available which has led to new insights into the underlying features of cancers. These include the identification of mutational signatures at both nucleotide and amino acid levels. Here, we discuss C > T transitions as a key nucleotide-level mutational signature that leads to a dramatic overrepresentation of arginine substitutions in cancers. We propose that this underlying C > T mutational signature canalizes possible arginine substitution outcomes, favoring histidine, cysteine, glutamine, and tryptophan. This initial asymmetry is then acted on at the amino acid level by purifying selection. Thus, a model of “sequential selection” could explain the documented bias towards arginine substitutions in multiple cancers.

**Abstract:**

Arginine is encoded by six different codons. Base pair changes in any of these codons can have a broad spectrum of effects including substitutions to twelve different amino acids, eighteen synonymous changes, and two stop codons. Four amino acids (histidine, cysteine, glutamine, and tryptophan) account for over 75% of amino acid substitutions of arginine. This suggests that a mutational bias, or “purifying selection”, mechanism is at work. This bias appears to be driven by C > T and G > A transitions in four of the six arginine codons, a signature that is universal and independent of cancer tissue of origin or histology. Here, we provide a review of the available literature and reanalyze publicly available data from the Catalogue of Somatic Mutations in Cancer (COSMIC). Our analysis identifies several genes with an arginine substitution bias. These include known factors such as IDH1, as well as previously unreported genes, including four cancer driver genes (FGFR3, PPP6C, MAX, GNAQ). We propose that base pair substitution bias and amino acid physiology both play a role in purifying selection. This model may explain the documented arginine substitution bias in cancers.

## 1. Introduction

Mutation is an essential feature of biology. It is the most important contributor to the cellular transformations that cause cancer and other diseases and is the primary source of variation acted on by evolution [1,2,3]. Point mutations caused by base pair substitutions, insertions, or deletions are common in human cancers and many other diseases [4]. Base pair substitutions alter the sequence of 64 different codons that code for the 20 amino acids. Point mutations can be silent (no amino acid change), missense (one amino acid is changed to another), nonsense (one amino acid is changed to a stop codon), or frameshifts (insertion or deletion of one or two base pairs).

Of the twenty amino acids, arginine appears to have a central role in gene expression, protein structure and function, and genome evolution. For example, arginine codons play a major role in determining the rate of protein translation [5,6,7], and the positive charge of the arginine side chain is critical for stabilizing protein tertiary structure [8,9]. Further, arginine is subject to a number of post-translational modifications including methylation, acetylation, ubiquitylation, citrullination, and mono-ADP-ribosylation, that impact a wide range of cellular processes such as epigenetics, signal transduction, and DNA damage response [10,11,12,13,14,15]. At the evolutionary level, differences in usage of the six arginine codons can be used as a species classification tool across the three domains of life [16]. Finally, in human cancers, the CGA arginine codon is most frequently mutated to a stop codon (nonsense) [17]. Thus, arginine is arguably one of the most important amino acids in biology.

In the last 10–15 years, numerous analyzed cancer genomes have been made available to the public. These include projects such as The Cancer Genome Atlas (TCGA), The Sanger Cancer Genome Project, and The Cell Lines Project. Genomes have also been made available through various user-friendly databases and collaborations such as the Catalogue of Somatic Mutations in Cancer (COSMIC) and the International Cancer Genome Consortium (ICGC), which compile these data and link them to independent studies from the literature [18,19,20]. This has allowed a largely unbiased analysis of mutation patterns in cancer cells. One observation is that arginine is the most frequently mutated amino acid in human cancers, with a tendency towards arginine loss [21]. In this paper, we review key findings in the literature and provide independent validation and additional data supporting some of these observations.

## 2. Materials and Methods

### Data Processing

A file with arginine mutations in all cancer tissues was downloaded as an excel file (.csv) from the COSMIC database (https://cancer.sanger.ac.uk/cosmic accessed on: 1 May 2021, version 94, hg38). For this analysis, only point mutations such as missense, nonsense, and silent mutations were studied and included in the working dataset. COSMIC provides data fields such as chromosome number, genomic position, mutated amino acid residue, and the specific nucleotide change. However, the arginine codon that was mutated, and the codon of the resulting mutated amino acid residue are not provided in COSMIC. Codon information for each point mutation was retrieved from Ensembl using the Newman application program interface (API) requesting program. The code for this program is included in Figure S1, and can be accessed through GitHub repository with additional documentation (https://github.com/devinelakurti/Newman-API-Requesting-Program/tree/main deposited on: 2 November 2021).

**Figure 1:**
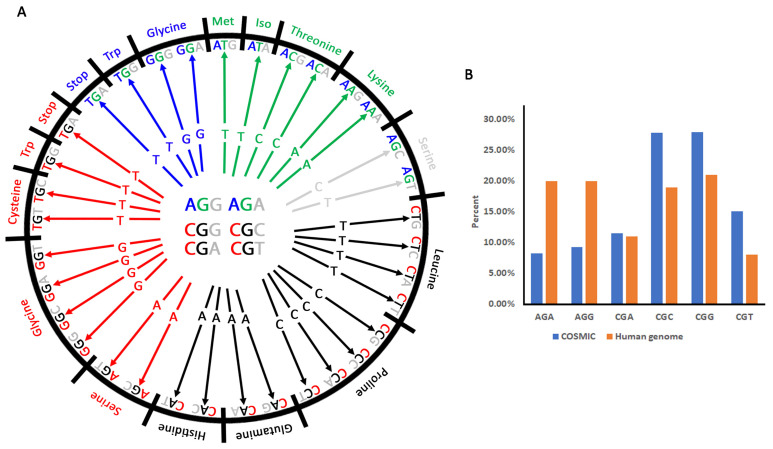
Possible amino acid substitutions from the six arginine codons. (**A**) Diagram showing base pair changes of the six arginine codons that can lead to amino acid substitutions and stop codons. Each arrow points to a base pair change within the six codons and its corresponding amino acid substitution. For simplicity, each base of the six codons is color-coded to correspond with the arrow. For example, if the first red C base of the **CGG** codon is changed to a **T**, it will produce a **TGG** codon which substitutes tryptophan for arginine. Note that some substitutions are more likely than others. Synonymous substitutions are not shown. (**B**) Arginine codon usage in human cancer and non-cancer cells. The graph shows all the reported arginine substitutions in cancer cells on COSMIC (765,956 counts in 69,455 unique cancer samples, blue bars) compared with observed arginine codon usage in human non-cancer cells (orange bars) as reported on GenScript (https://www.genscript.com/tools/codon-frequency-table, accessed on 5 November 2021). Values are plotted as a percentage of each codon usage compared with total usage. These data largely agree with [22].

Figure S2 illustrates the details of the retrieval process of the arginine codon using the specific data fields given in COSMIC. This schematic illustrates how the data fields provided in COSMIC (chromosome number and genomic position), and the calculated position of mutations, were translated into genomic position ranges of codons. For instance, variable *x* in the genomic position range represents the genomic position of the point mutation. Mutation sense determines whether the output codon from the API requires analysis of the reverse and complement. Genomic position ranges are thus determined both by whether mutations fall on the Watson or the Crick strand and the position of the mutation in the arginine triplet codon. Based on the retrieved arginine codon, the specific nucleotide change, and the mutated amino acid residue, a program was developed to automate the process of identifying the codon of the resulting mutated amino acid (Figure S1). The logic of this program is based on possible changes of all arginine codons (Figure 1A, Table S1). After the retrieval of both the arginine codon and codon of the resulting mutated amino acid, both sets of information were integrated into the rest of the COSMIC dataset for further analyses. We also identified genes with a clear skew towards cysteine, histidine, glutamine, or tryptophan. Genes were called “skewed” if a minimum of 60% of all arginine substitutions produced one amino acid (e.g., histidine) at the expense of the others. To minimize statistical aberrations, genes with fewer than 40 independent tumor samples contributing to this skew were excluded.

To calculate the control percentages reported in Figure 3, all point mutations reported in COSMIC Mutation were compiled in a dataset called coding control. All silent mutations were filtered out to create a separate dataset called silent coding control. Coding control percentage for each amino acid was calculated with the numerator being the number of point mutations that resulted in that particular amino acid and the denominator is all the coding point mutations reported in the coding control dataset. Similarly, silent coding percentage for each amino acid was calculated with the numerator being the number of silent mutations that code for a particular amino acid and the denominator is all the silent mutations reported in the silent coding control dataset. Additionally, another dataset called “non-coding control” was compiled with all point mutations from the “noncoding variants” data in COSMIC. With this noncoding control dataset, control percentages were calculated for each nucleotide change. The numerator is the number of point mutations with the specific nucleotide change of interest and the denominator is the total number of noncoding point mutations in the dataset. All data were analyzed in IBM SPSS, v27.

## 3. Results

### 3.1. Non-Synonymous Substitution Bias of Arginine Codons

Six synonymous codons are used for arginine (Figure 1A), and base pair substitutions in these codons can generate twelve different amino acids (not including synonymous changes) and a stop codon. In addition, arginine is one of only two amino acids for which substitutions in the first codon position can result in synonymous change (the other is leucine). There are 54 possible substitutions from the six arginine codons. Four codons (AGA, AGG, CGA and CGG) can produce synonymous substitutions from mutations in the first position (Table S1). All other synonymous changes result from mutations in the third position. However, in mutations leading to amino acid substitution, over 75% of all arginine substitutions occurring in a cancer context are histidine, cysteine, tryptophan, or glutamine [21]. Interestingly, this skew also resembles evolutionary mutation profiles for arginine [23], suggesting similar selection biases operating in both cancer and evolutionary (speciation) contexts. These findings point to a non-random pattern of amino acid substitutions in human cancers [24].

Organisms commonly display preferences for certain synonymous codons over others [25,26,27] (Figure 1B). This codon usage bias has a major role in gene expression, regulating translation speed and protein folding [28,29,30], as well as mRNA structure, processing, and stability [31,32,33,34]. Additionally, in cancer cells, codon usage is optimized to accommodate high translation of cell cycle regulatory genes [35]. Codon usage bias is species-specific [36] with biases in arginine usage correlating with speciation [16]. In humans, four codons (AGA, AGG, CGG, CGC) are each used approximately 20% of the time whereas two (CGA and CGT) are used only ~10% of the time (Figure 1B). In vertebrates, an increased preference for G/C-ending codons (base at third position) correlates with an increase in G/C bias across the genome [37,38]. With the exception of AGA, arginine codons generally follow this pattern. For instance, mutations in the CGC, CGG, and CGT codons are most likely to substitute arginine for another non-synonymous amino acid and previous analyses of COSMIC v78 (~18,000 cancer samples) show these three codons (CGC, CGG, CGT) and a fourth (CGA) account for most arginine substitution biases [22]. Our present analyses of COSMIC v94, which contains over 68,000 samples, came to a similar conclusion (Figure 1B), supporting the idea that this observation reflects a biological rather than a technical bias. Remarkably, we also find that CGC, CGG, and CGT are three of the four most likely codons to generate synonymous arginine substitutions (the other is CGA) (Table S1).

Molecular evolution of genomes was initially proposed to occur through a combination of neutral evolution and genetic drift [39,40,41]. This theory postulates that most deleterious mutations are eliminated by natural selection whereas genetic drift fixes mainly neutral mutations that do not drastically change the phenotype. Conversely, fixation of mutations that greatly change the phenotype is very rare. Other models have argued that substitution is subject to a combination of purifying (or negative) selection which eliminates deleterious mutations and positive selection which promotes fixation of beneficial mutations. Page and Holmes argue that these two models are distinct, as evolution would occur by chance with neutral selection and by necessity with purifying selection [42]. The substitution bias of arginine amino acids in both cancers and evolution support a model in which purifying selection drives the evolution of human cancer genomes. In practice, this would mean that certain amino acids are not tolerated when substituted in the wild-type position of arginine [43], and are subsequently “purified” or eliminated. Thus, the only observable mutations in the population would be ones which replaced arginine with a tolerated amino acid.

This analysis, in conjunction with the fact that four of the six arginine codons account for most mutations, indicates that the increased frequency of non-synonymous amino acid substitutions of arginine in human cancers is not merely a statistical consequence of usage bias or the mutation possibility of its six codons. Instead, it suggests that it is an outcome of selection on specific amino acid substitutions in key codons that promote cellular transformation and cancer progression. Arginine substitutions in human cancers thus appear to be driven by purifying selection rather than neutral selection.

### 3.2. Arginine Substitutions in Human Cancers Are Driven Mainly by C/G > T/A Transitions

**Figure 2:**
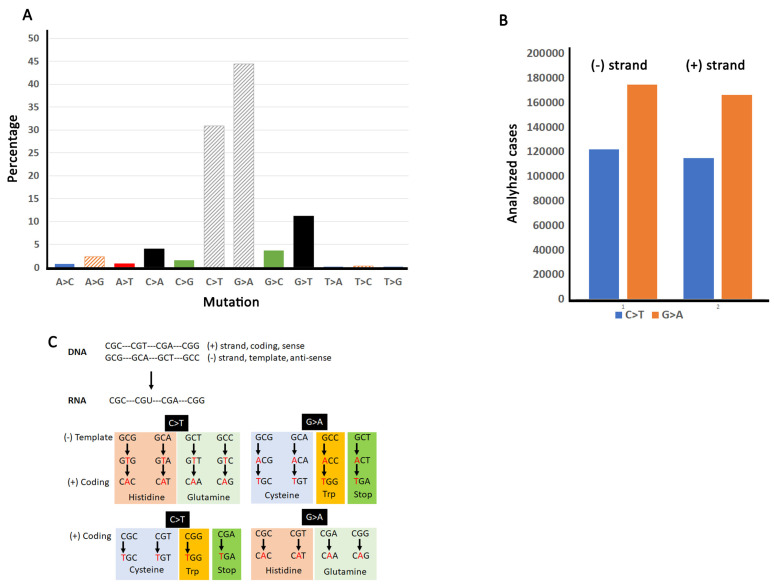
C/G > T/A transitions are responsible for over 75% of all arginine substitutions. (**A**) Percent of each base pair change in the COSMIC V94 (our analysis). For this analysis, the six COSMIC signatures were split into 12 to show opposite strand mutations. (**B**) Strand specific frequencies of C > T and G > A changes. (**C**) Four arginine codons may be responsible for most arginine substitutions. Note that if we consider G > A to be the same as C > T, it is possible to envision how most arginine substitutions could be generated by a C > T transition. In this model, the G > A would become C > T within one round of DNA replication; therefore, the two would look like the same mutation.

Base pair changes fall into two general categories: transitions (purine-to-purine or pyrimidine-to-pyrimidine) and transversions (purine-to-pyrimidine or pyrimidine-to-purine) [44]. Despite twice as many possible transversions, most mutations that drive evolution are transitions [45,46,47]. A statistical study also showed that transitions outnumbered transversions in human evolution, at least since the divergence from rodents [23]. This parallels mutation signatures in cancers [48] and even quiescent cells [49] which have a higher burden of transitions than transversions. Further, mutation may accumulate independently of DNA replication, suggesting errors during cell division are not the only determinant of mutation [50]. Our analysis of COSMIC v94 shows that most base pair substitutions in cancer genomes are C > T and G > A (Figure 2A) and there is no strand bias for either C > T or G > A mutations (Figure 2B) which agrees with previous analyses [22].

Base pair substitution frequencies of the six arginine codons immediately reveal a selection preference for C > T and G > A [22]. The C > T substitution, in particular, is indicative of a selection bias, as C occurs in the first position of four of the codons (CGA, CGG, CGT, CGC; Figure 1A). Substitution of C for T in this first position produces stop (CGA to TGA), tryptophan (CGG to TGG), or cysteine (CGT to TGT; CGC to TGC). Note that a third position C > T mutation in CGC (to CGT) is silent (Figure 1A), which may explain why CGC is the most frequently mutated codon. However, the high frequency of CGG to TGG and CGT to TGT indicates a clear selection bias for tryptophan and cysteine, respectively, whereas the CGA to TGA mutation occurs very rarely because it introduces the stop codon [17]. G > A mutations can produce substitutions in all six codons. Remarkably, a high percentage of mutations convert the CGA codon to CAA (arginine to glutamine). The second most mutated codon is AGG which can be converted to lysine (AAG) or is silent (AGA). Mutations in the other codons produce histidine (CGC to CAC; CGT to CAT), glutamine (CGG to CAG), silent (CGG to CGA), or lysine (AGA to AAA) (Supplementary Table S1). G > A mutations are also most frequent for the AGA codon which results in isoleucine (AGA to ATA) or the AGG codon which results in methionine (AGG to ATG) or serine (AGG to AGT).

These analyses uncover a strong codon substitution bias, in which 75% of arginine substitutions are driven by C/G > T/A transitions in cancer genomes (Figure 2A and [22]). Remarkably, only four of the six arginine codons contribute to these substitutions (Figure 2C). These C/G rich codons permit C/G > T/A transitions that substitute arginine for four different amino acids (cysteine, glutamine, histidine and tryptophan). COSMIC lists six signatures rather than 12 [19,48,51] as the other half can be generated by mutations on the other strand. In other words, a C > T transition on one strand yields a G > A transition on the other strand, and when coupled with replication, both transitions generate the same mutation [52]. Certain cancers seem to show a bias for coding vs. non-coding strands, as suggested by other studies [49]. However, as our analyses compile data across all cancers, this bias is not obvious.

Instead, our analyses indicate a bias for both C > T and G > A transitions in coding regions (Table S2). Specifically, we find approximately twice as many transitions in coding regions (30.88% for C > T and 44.44% for G > A) over non-coding regions (16.73% for C > T and 17.02% for G > A). In addition, these transitions are more likely to occur within arginine codons than other codons (30.88% arginine vs. 25.18% total for C > T; 44.44% arginine vs. 26.49% total for G > A).

C > T transitions can be produced by deamination of CpG sites [53]. Indeed, mutational signatures due to deamination have been identified in cancer cells [48]. “Clock-like” mutational signatures (i.e., mutations that occur during the lifetime of a cell irrespective of its identity) appear to be a major producer of C > T transitions in cancer cells [51]. However, a study in yeast found that decreased processivity of polymerase delta resulted in primarily C > T transitions, suggesting several mutation processes may be at work [54]. G > A transitions, on the other hand, can be produced by guanine oxidation [22,52]. Regardless of mechanism, we do not find a strand bias for either C > T or G > A when compiling data for all cancers (Figure 2B), which largely agrees with previous findings [22].

### 3.3. Purifying Selection at the Amino Acid Level May Be Strongly Biased by Selection at the Nucleotide Level

It has been observed that amino acid substitutions in cancer cells are not completely random [21,22,43,55,56]. These analyses revealed that arginine mutations in cancer genomes are strongly biased towards cysteine, glutamine, histidine, and tryptophan. Given that 33% of base pair substitutions in arginine are synonymous (Table S1), a silent mutation should have been the most frequently observed change. Instead, arginine synonymous substitutions are found with approximately three-fold lower frequency than predicted [22]. One argument for the bias in arginine mutations, particularly the most prominent Arg > His mutation, is that these mutations are adapted to the elevated pH in cancer cells [57].

**Figure 3:**
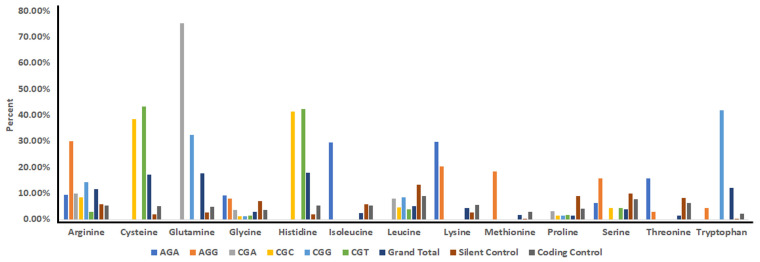
Observed distribution of nucleotide changes and amino acid substitutions in human cancers from the six arginine codons. We re-analyzed the COSMIC data (version 94) to determine the contribution of each arginine codon to arginine amino acid substitutions. The graph is a composite showing both the amino acid substitutions and the base pair changes resulting in those substitutions. Included as controls are the COSMIC frequency of changes that result in silent mutations, those that result in amino acid substitutions on the non-coding strand, and any change occurring in untranscribed regions, as reported on COSMIC. “Grand total” represents the percentages of nucleotide changes present in arginine mutations regardless of the starting or mutated codon.

However, an analysis of the arginine codons responsible for this amino acid bias reveals that they are CGC and CGT (cysteine), CGC and CGT (histidine), CGA and CGG (glutamine), and CGG (tryptophan) (Figure 3). Arginine substitutions to each of these amino acids are possible from two different codons (Table S1), and cysteine and histidine have a roughly equal chance of being generated from either codon. Interestingly, this is not the case for glutamine and tryptophan. Our reanalysis, which takes into consideration individual codons, found that for glutamine, substitutions from the CGG codon occur at less than half the frequency of the CGA codon (Figure 3). This reveals strong selection for CGA-driven glutamine substitutions, especially considering the genome usage bias of the CGA codon is half that of the CGG codon (Figure 1B). Similarly for tryptophan, only ~10% of the substitutions are due to mutations of the AGG codon despite virtually no difference in genome usage biases of AGG vs. CGG. Taken together, these data indicate that mutation bias occurs first at the base pair level (i.e., nucleic acid and codon) followed by potential purifying selection at the amino acid level.

### 3.4. Cancer or Gene Specific Arginine Mutation Bias

Arginine depletion is common to all cancers and is a hallmark of multiple tumor suppressors [22]. However, cancer types can be characterized by different mutational signatures [48,58], with some showing strong and specific biases towards certain amino acid substitutions. For instance, many cancer types show a clear arginine to histidine bias [55], occurring in both tumor suppressor and non-tumor suppressor proteins. One excellent example of this is gliomas, which show a strong preference for the R132H mutation of isocitrate dehydrogenase (IDH1; [56]). This particular substitution produces a metabolic byproduct that appears to increase the oncogenic potential of gliomas by interfering with histone demethylases and increasing oxidative species-related DNA damage [59]. Perhaps counterintuitively, the presence of this mutation is associated with better prognoses compared with glioma patients with a wildtype IDH1 [60,61,62,63]. This appears to be related to the low NADPH production levels in IDH1 mutant cells which renders patients sensitive to therapy [64].

We generated a complete list of genes showing skews in arginine substitution biases for histidine, cysteine, glutamine, and tryptophan (Table 1 and Table S3; see Materials and Methods for criteria). We classified genes as driver and non-driver based on a recently published characterization [65]. Under this classification, IDH1 is a driver gene. How can this be reconciled with the fact that IDH1 mutations are associated with favorable prognosis? It appears that if IDH1 mutations occur early, they have an adverse effect on DNA damage repair [66], as well as other cellular transformation processes [59], including TERT reactivation [67] and chromatin remodeling [68]. Our analysis also identified four other driver genes (FGFR3, PPP6C, MAX, GNAQ; Table 1). Fibroblast growth factor receptor 3 (FGFR3) is a well-established cancer driver gene in several cancers, and single molecule inhibitors of this gene are used as therapeutic agents [69]. Protein phosphatase 6 (PP6 encoded by PPP6C) encodes the catalytic subunit of a PP2A like phosphatase [70], a molecular regulator of RAS and other RAS associated pathways (e.g., BRAF/MEK/ERK) involved in cell proliferation [71]. PP6 participates in many processes including DNA damage repair, inflammation, and the immune response, and PP6 mutations are associated with tumor progression [72]. MAX is a cofactor of MYC and other MYC-related transcription factors involved in cell proliferation [73,74]. MAX has tumor-suppressive functions [75]. GNAQ encodes the alpha subunit of a heterotrimeric G-protein and mutations are associated with certain melanoma cancers [76].

Skewed genes include a number of additional factors with established roles in cancer (e.g., BMP8A and BUB1 [77,78]), as well as others (Table 1, Table S3). The candidates identified in this study did not show any obvious protein class preferences (e.g., kinases versus transcription factors). Indeed, skewed genes impact a wide range of cellular processes such as intracellular signaling, cytoskeletal architecture, metabolism, and mitosis (Table 1, Table S3). Arginine depletion in cancers thus appears to target genes that are likely to increase the transformation and proliferative potential of cells. In addition, our analyses identify a number of other poorly characterized genes (e.g., OR1L6 or OR4CC3) which may be high-confidence candidates to modulate tumor progression, proliferation, and/or metastasis.

## 4. Conclusions and Perspectives

The combination of thousands of publicly available cancer genomes and advanced computational techniques has enabled unprecedented insight into common and distinct features of cancers. These include mutational signatures at the nucleotide level and skews or biases at the amino acid level. For instance, multiple studies, including this one, identify C > T transitions as the dominant mutational signature underlying the dramatic overrepresentation of arginine substitutions in cancers. We propose that these two features are linked. Specifically, an underlying C > T mutational signature canalizes possible arginine substitution outcomes, creating an initial asymmetry in favor of histidine, cysteine, glutamine, and tryptophan. Purifying selection acting at the amino acid level then reinforces this asymmetry, which can occur in a protein- or tissue-dependent manner. For example, stomach cancers show a pronounced Arg > His bias, whereas skin cancers have a strong Arg > Cys bias [35]. Determining why such context-dependent behaviors happen, and whether this model of “sequential selection” extends to other signatures, are important next steps for the field.

## Figures and Tables

**Table 1:** Substitution bias of selected genes ^1^.

^2^ Gene	Most Frequent Substituted Residue				Most Frequent Substituted Nucleotide		^3^ Driver Gene
	Cysteine	Glutamine	Histidine	Tryptophan	C > T	G > A	Yes/No
**IDH1**			**76%**			**76.36%**	**YES**
TCP10L2			61%			68.97%	NO
NEK9			74%			86.90%	NO
TXK			60%			83.13%	NO
CYP2D6			69%			68.66%	NO
NCF1			76%			76.47%	NO
OR4C3			70%			76.60%	NO
KRTAP4-8			85%			86.96%	NO
BMP8A			78%			77.78%	NO
**FGFR3**	**80%**				**88.59%**		**YES**
RFPL3	66%				71.26%		NO
**PPP6C**	**63%**				**66.67%**		**YES**
HASPIN	73%				76.54%		NO
DTX2	63%				66.10%		NO
PRSS1	77%				79.25%		NO
POTEB2	73%				73.08%		NO
CAMKK2	64%				74.00%		NO
PARN	69%				73.81%		NO
OR9G1	73%				73.17%		NO
NPIPA5	80%				80.00%		NO
PRB2		94%				94.94%	NO
BUB1B		70%				77.05%	NO
AC004223.3		88%				92.56%	NO
RAD51D		89%				93.04%	NO
GNL3		93%				93.58%	NO
RNASEL		69%				79.31%	NO
**MAX**		**68%**				**75.86%**	**YES**
**GNAQ**		**64%**				**76.47%**	**YES**
IRF5		62%				82.72%	NO
CS		80%				81.36%	NO
PRB1		91%				93.48%	NO
FAM120B		70%				76.74%	NO
CLEC4M		76%				87.80%	NO
OR1L6		68%				75.00%	NO
IST1		75%				82.50%	NO
PDCL3				61%	71.74%		NO
SPAG11B				71%	76.19%		NO

^1^ The table colors correspond to arginine substitution biases towards the four amino acids (cysteine, histidine, glutamine and tryptophan). ^2^ Only genes with a substitution bias over 60% are shown. Please see Supplementary Table S3 for further details. ^3^ Genes in bold are characterized as driver by Martinez-Jimenez et al. [65].

## Data Availability

Data were obtained from the COSMIC database, which is freely available for non-commercial users.

## Supplementary Materials

The following are available online at https://www.mdpi.com/article/10.3390/cancers13246274/s1. Figure S1: Newman API requesting program, Figure S2: Arginine codon retrieval process, Table S1: Potential amino acid substitutions from mutations in the first, second and third position in any of the six arginine codons, Table S2: Base pair substitution bias in the six arginine codons. Table S3: Genes showing clear biases in arginine substitution towards histidine, cysteine, glutamine, or tryptophan.
