# Supplementary material for: Arginine Depletion in Human Cancers
Source: Cancers (Basel). 2021 Dec 14;13(24):6274. doi: 10.3390/cancers13246274 (PMC8699593; doi:10.3390/cancers13246274)
Supplement: Supplementary file 1 [file cancers-13-06274-s001.zip › Supplementary Figure S1.pdf]

Supplementary Figure S1. Newman API Requesting Program.

```
----------------------
How to install Newman:
----------------------

If you have npm installed, run this command in your command-line interface (CLI) "npm install -g newman"

If you don't have npm installed,

Download and install Node.js.
Then, run the command "npm install -g newman" in your CLI.



----------------------
Command Line Arguments
----------------------
newman run <postman collection json file name> -d <input file name > --delay-request <how much delay between the requests> >> <output file>

Example commmand:
newman run DNATest.postman_collection_Ensembl.json -d "test.csv" --delay-request 10 >> testfinal.out



-------------------------
Issues
-------------------------

Q.  File not found error postman collection json file , input file and  output?

A.  Better to keep all these files in the same directory to avoid this issue.

Q.  How to keep the input files "postman collection json file , input file and  output"?

A.  In case if you want to keep input files in different directories then refer them with full path or absolute path

e.g  if you keep all the input files in the directory "D:\testing\" then you should refer those input files as below
newman run D:\testing\DNATest.postman_collection_Ensembl.json -d "D:\testing\test.csv" --delay-request 10 >> D:\testing\testfinal.out
```


```
{
    "info": {
        "_postman_id": "a675aa41-164a-4eb5-904f-75364a4f1a14",
        "name": "DNATest",
        "schema": "https://schema.getpostman.com/json/collection/v2.1.0/collection.json"
    },
    "item": [
        {
```

```
      "name": "getData",
      "request": {
        "method": "GET",
        "header": [
          {
            "key": "Content-Type",
            "value": "text/x-fasta",
            "type": "text",
            "disabled": true
          }
        ],
        "url": {
          "raw": "https://rest.ensembl.org/sequence/region/human/{{genome}}?content-type=text/x-fasta",
          "protocol": "https",
          "host": [
            "rest",
            "ensembl",
            "org"
          ],
          "path": [
            "sequence",
            "region",
            "human",
            "{{genome}}"
          ],
          "query": [
            {
              "key": "content-type",
              "value": "text/x-fasta"
            }
          ]
        }
      },
      "response": []
    }
  ],
  "event": [
    {
      "listen": "prerequest",
      "script": {
        "type": "text/javascript",
        "exec": [
          ""
        ]
      }
    },
    {
      "listen": "test",
```

```
        "script": {
          "type": "text/javascript",
          "exec": [
            "reponseBodyData = responseBody;",
            "pm.test(\"Response Body Printout= (\" + responseBody",
            " + \")\", function () {",
            "});"
          ]
        }
      }
    ],
    "variable": [
      {
        "key": "genome",
        "value": "{{CHR#}}:{{First}}..{{Third}}:1"
      }
    ]
}
```


Supplementary Figure #?: Figuring out resulting mutated amino acid

```python
from openpyxl import load_workbook
wb = load_workbook('Arginine_ExtractedData.xlsx')
lookup = wb['ReferenceSheet']
data = wb["cosmic_complete_mutation_argini"]
finalvalue="";

for row in data.iter_rows():
    for newrow in lookup.iter_rows():
            if row[10].value == newrow[0].value and row[14].value == newrow[1].value:
                if row[11].value==None:
                    row[11].value=newrow[2].value;
                    row[12].value=newrow[3].value;
                elif row[11].value!=None and row[13].value==None:
                    row[13].value=newrow[2].value;
                    row[14].value=newrow[3].value;
                elif row[13].value!=None and row[15].value==None:
                    row[15].value=newrow[2].value;
                    row[16].value=newrow[3].value;
                    print("idk")
                else:
                    row[17].value=newrow[2].value;
                    row[18].value=newrow[3].value;
    wb.save('Arginine_CodonsInserted.xlsx')
```

Supplementary Figure #?: Partitioning Control Data

Coding Control

```
#!/usr/bin/env python
# coding: utf-8

# In[2]:


import pandas as pd
import pyreadstat
import numpy as np

df, meta = pyreadstat.read_sav("C:/Users/nelakurti.1/Downloads/Mutation coding.sav")

ap=(df['MutationDescription'].values == '')
count = np.count_nonzero(ap)
print(count)
[i for i, x in enumerate(ap) if x] #BEFORE
df.head()

df['ExtractedCDS'] = df['MutationCDS'].str[-3:] #extracts _>_ from MutationCDS to ExtractedDS
df['OG_AA'] = df['MutationAA'].str[2:] # extracts the characters after p. in MutationAA
df['OG_AA'] = df['OG_AA'].str.replace('\d+', '') #removes any and all digits in the extracted verison of MutationAA
df['Mutated_AA'] = df['OG_AA'].str[-1:] #seperates the two digits
df['OG_AA'] = df['OG_AA'].str[:-1] #seperates the two digits

ap=(df['MutationDescription'].values == '')
count = np.count_nonzero(ap)
print(count)
[i for i, x in enumerate(ap) if x] #AFTER
df.head()


# In[7]:


ExtractedCDS_counts = df.groupby(['MutationDescription', 'ExtractedCDS'])[['ExtractedCDS']].count()
print(ExtractedCDS_counts)
ExtractedCDS_counts.to_csv("C:/Users/nelakurti.1/Downloads/ExtractedCDS_counts.csv")


# In[11]:


df.OG_AA.unique()
OG_AA_counts = df.groupby(['MutationDescription', 'OG_AA'])[['OG_AA']].count()
print(OG_AA_counts)
OG_AA_counts.to_csv("C:/Users/nelakurti.1/Downloads/OG_AA_counts.csv")
```

```
# In[15]:


df['Mutated_AA'] = np.where((df['Mutated_AA'] == 'OG_AA'), #Identifies the case to apply to
                    df['OG_AA'],      #This is the value that is inserted
                    df['Mutated_AA'])      #This is the column that is affected
df.head()


# In[16]:


MutatedAA_counts = df.groupby(['MutationDescription', 'Mutated_AA'])[['Mutated_AA']].count()
print(MutatedAA_counts)
MutatedAA_counts.to_csv("C:/Users/nelakurti.1/Downloads/MutatedAA_counts.csv")
```

Noncoding Control

```
#!/usr/bin/env python
# coding: utf-8

# In[1]:


import pandas as pd
import numpy as np
import pyreadstat

df, meta = pyreadstat.read_sav("C:/Users/nelakurti.1/Downloads/Mutation non coding.sav") #creates a pandas
dataframe

ap=(df['MutationDescription'].values == '')
count = np.count_nonzero(ap)
print(count)
[i for i, x in enumerate(ap) if x] #BEFORE
df.head()

df['ExtractedCDS'] = df['MutationCDS'].str[-3:] #extracts _>_ from MutationCDS to ExtractedDS
df['OG_AA'] = df['MutationAA'].str[2:] # extracts the characters after p. in MutationAA
df['OG_AA'] = df['OG_AA'].str.replace('\d+', '') #removes any and all digits in the extracted verison of MutationAA
df['Mutated_AA'] = df['OG_AA'].str[-1:] #seperates the two digits
df['OG_AA'] = df['OG_AA'].str[:-1] #seperates the two digits

ap=(df['MutationDescription'].values == '')
count = np.count_nonzero(ap)
print(count)
[i for i, x in enumerate(ap) if x] #AFTER
df.head()
```

```
# In[9]:


ExtractedCDS_counts = df.groupby(['MutationDescription', 'ExtractedCDS'])[['ExtractedCDS']].count()
print(ExtractedCDS_counts)
ExtractedCDS_counts.to_csv("C:/Users/nelakurti.1/Downloads/NonCodingExtractedCDS_counts.csv")
```

Silent Control

```
#!/usr/bin/env python
# coding: utf-8

# In[1]:


import pandas as pd
import numpy as np
import pyreadstat

df, meta = pyreadstat.read_sav("C:/Users/nelakurti.1/Downloads/Mutation coding.sav") #creates a pandas dataframe

ap=(df['MutationDescription'].values == '')
count = np.count_nonzero(ap)
print(count)
[i for i, x in enumerate(ap) if x] #BEFORE
df.head()

df['ExtractedCDS'] = df['MutationCDS'].str[-3:] #extracts _>_ from MutationCDS to ExtractedDS
df['OG_AA'] = df['MutationAA'].str[2:] # extracts the characters after p. in MutationAA
df['OG_AA'] = df['OG_AA'].str.replace('\d+', '') #removes any and all digits in the extracted verison of MutationAA
df['Mutated_AA'] = df['OG_AA'].str[-1:] #seperates the two digits
df['OG_AA'] = df['OG_AA'].str[:-1] #seperates the two digits

ap=(df['MutationDescription'].values == '')
count = np.count_nonzero(ap)
print(count)
[i for i, x in enumerate(ap) if x] #AFTER
df.head()


# In[5]:


Silent = df[df['Mutated_AA'] == '=']
Silent_counts = Silent.groupby(['MutationDescription', 'OG_AA'])[['OG_AA']].count()
print(Silent_counts)
```

```
Silent_counts.to_csv("C:/Users/nelakurti.1/Downloads/SilentAA_counts.csv")


# In[ ]:
```
