# Supplementary material for: Arginine Depletion in Human Cancers
Source: Cancers (Basel). 2021 Dec 14;13(24):6274. doi: 10.3390/cancers13246274 (PMC8699593; doi:10.3390/cancers13246274)

Supplementary Figure S2. Arginine codon retrieval process. Codon information for each point mutation was retrieved from Ensembl using application program interface (API). For accurate retrieval of the codon, the API required the fields: chromosome number and genomic position range for the specific genomic sequence. This schematic illustrates how given the data fields (chromosome number, mutation sense, and genomic position) provided in COSMIC and calculated position of mutation was translated to figure out the genomic position range of the codons. Based on the mutation sense and position of mutation, the genomic position range expressions differ. The variable X in the genomic position range expressions represent the genomic position of the point mutation provided in COSMIC. The mutation sense determines whether the output codon from the API needs to be reversed and complemented.

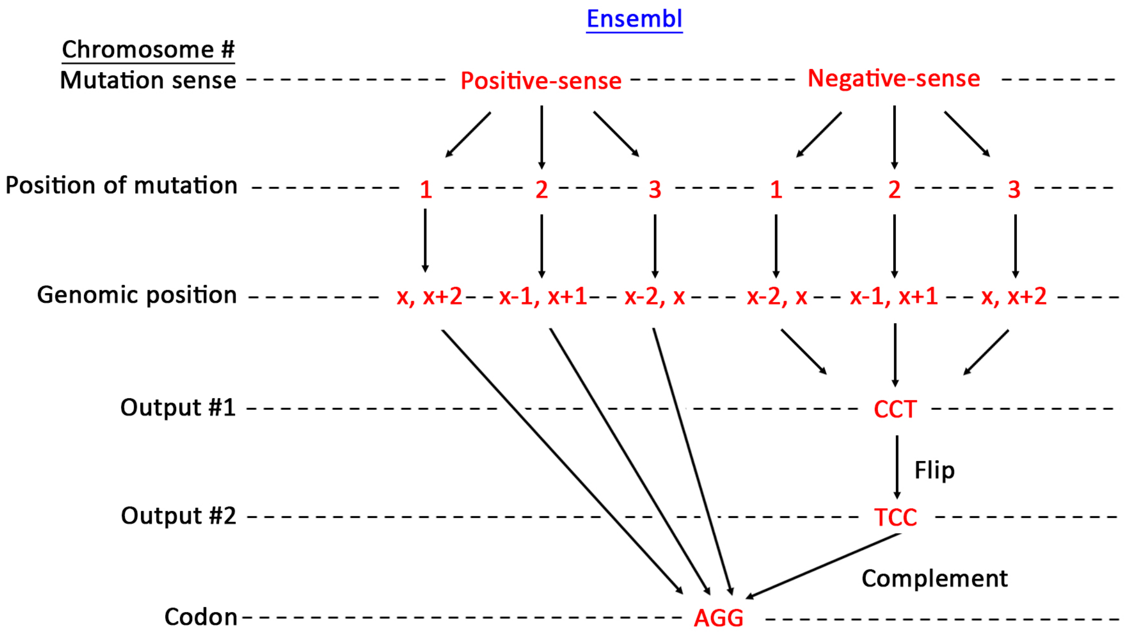

Supplement: Supplementary file 1 [file cancers-13-06274-s001.zip › Supplementary Figure S2.pdf]
